# Exploring Ascorbic Acid's Role in Orthopedic Practices: Present Theories, Innovative Approaches, and Prospects

**DOI:** 10.7759/cureus.60164

**Published:** 2024-05-12

**Authors:** Rayed Qamar, Raghvendra Choubisa, Akshit Sen, Mit Parikh, Siddharth Bishnoi, Mayank Yadav, Shubham S Srivastava, Haseeb S Sayed, Chandresh Choudhary

**Affiliations:** 1 Orthopedics and Traumatology, Geetanjali Medical College and Hospital, Udaipur, IND; 2 Orthopedics, Geetanjali Medical College and Hospital, Udaipur, IND; 3 Orthopedic Surgery, Geetanjali Medical College, Udaipur, IND; 4 Orthopedics, Geetanjali Medical College and Hospital, Jaipur, IND

**Keywords:** tendon healing, osteoarthritis, crps, bone healing, ascorbic acid

## Abstract

In the human body, ascorbic acid (AA) is known for its potent antioxidant and reducing properties and also plays a vital role in supporting the growth of bones and cartilage. It has been used extensively in orthopedic surgery. Ongoing studies under the umbrella of ascorbic acid research investigate its impact on bone and tendon physiology, as well as its influence on joint replacement and postoperative pain. The majority of both laboratory and human studies link the usage of ascorbic acid to enhanced bone health and improved tendon healing. Recent literature suggest that ascorbic acid administration may have a positive impact on the outcome of orthopedic procedures. On the other hand, controversy exists regarding the efficacy of ascorbic acid in reducing the incidence of complex regional pain syndrome. In brief, the effectiveness of ascorbic acid in enhancing orthopedic procedure outcomes remains a subject of ongoing investigation. Although certain studies have hinted at the potential positive influence of ascorbic acid on these outcomes, further research is required to validate its effectiveness and ascertain the ideal dosage and method of administration for maximizing its anticipated advantages. To establish the efficacy of ascorbic acid in improving orthopedic procedure outcomes, rigorous human trials of high quality are imperative. The aim of this review was to provide an overview of ascorbic acid's utilization in orthopedic practices and to pinpoint prospective areas for future research.

## Introduction and background

Reactive oxygen species (ROS), natural byproducts of regular metabolism, play a crucial role in maintaining balance within the body and aiding cellular communication [1]. However, when ROS production surpasses the body's antioxidant defenses, it can lead to protein oxidation, lipid peroxidation, and damage to nucleic acids [2]. In their research, Zhou et al. discovered that scavengers targeting ROS exhibited strong analgesic effects in a rat model [3]. An imbalance between ROS production and the body's antioxidant capacity is believed to hinder bone healing after a fracture [4]. Choi et al.'s study revealed that ascorbic acid (AA) enhances the formation of bone-building cells (osteoblastogenesis) while simultaneously suppressing bone-resorbing cells (osteoclastogenesis) in living organisms [5]. Additionally, ascorbic acid has been found to possess various preventive properties against the progression of osteoarthritis (OA), such as reducing cell death, inflammatory cytokines, and matrix metalloproteinase (MMP) expression, aside from its well-known antioxidative role [6]. Observational data support the notion that a high dietary intake or supplementation of ascorbic acid may lower the risk of hip fractures in postmenopausal females [7]. The evidence indicating a decreased occurrence of complex regional pain syndrome after treating distal radius fractures with adjuvant ascorbic acid has sparked considerable discussion and has garnered moderate support from the American Academy of Orthopaedic Surgeons [8]. Given its potential benefits, affordability, and safety profile, orthopedic surgeons should consider incorporating ascorbic acid supplementation into the treatment of various musculoskeletal injuries [8].

The aim of this review was to outline the role of ascorbic acid in orthopedic practices and to pinpoint potential areas for future investigation.

## Review

Perioperative pain management

Postoperative pain can heighten the likelihood of complications, prolong hospital stays, and delay the return to regular activities. A study by Chen et al. suggested moderate-level evidence supporting the use of a 2 g preoperative dose of vitamin C (VC) as an adjunct for reducing postoperative morphine consumption and high-level evidence supporting perioperative vitamin C supplementation of 1 g/day for 50 days for type 1 complex regional pain syndrome (CRPS-I) prevention after extremity surgery [9]. Opioids, such as morphine, which act on the mu opioid receptor, are commonly relied upon for treating moderate to severe pain and exhibit effectiveness in these scenarios. However, they come with a range of undesirable effects, including respiratory depression, constipation, immune suppression, and, with prolonged use, the development of tolerance, dependence, and the potential for abuse [10]. Nonsteroidal anti-inflammatory drugs (NSAIDs) are another option for managing postoperative pain, but they carry risks such as peptic ulcers and gastroesophageal reflux disease (GERD) [11].

CRPS

Type 1 complex regional pain syndrome (CRPS) is a severe condition marked by severe pain, swelling, and instability of blood vessels. It typically develops after an injury or surgery involving an extremity, posing a significant challenge in orthopedic practice. Its prevalence is noteworthy, ranging from 10% to 22% after wrist fractures and 10% following foot and ankle surgeries. While the exact underlying mechanism of CRPS remains unclear, it is believed that the dysregulation and increased permeability of blood vessels induced by free radicals play a key role. The potential of ascorbic acid in preventing the onset of CRPS has been explored in a limited number of studies.

In a double-blind trial, Zollinger et al. conducted a randomized study involving 127 conservatively treated distal radius fractures. The participants were randomly assigned to receive either 500 mg of ascorbic acid or a placebo daily for 50 days, beginning from the day of the injury [12]. The incidence of CRPS, evaluated using diagnostic criteria proposed by Veldman et al., was 22% in the placebo group and notably lower at 7% in the ascorbic acid group at the one-year follow-up [13]. The 95% confidence interval for the difference ranged from 2% to 26%. Notably, complaints during cast wear and the type of fracture emerged as factors increasing the risk of CRPS development.

More recently, Laumonerie et al. conducted a retrospective comparative study to examine whether oral ascorbic acid (VC) supplementation is linked to a reduced risk of type 1 complex regional pain syndrome (CRPS-I) following subacromial shoulder surgery (SaSS) [14]. In group 1, consisting of 266 patients, no oral VC supplementation (500 mg) was provided. Conversely, group 2 included 267 patients who received 1 g of VC daily for 45 days, starting from the day of the surgery. Patient evaluations were conducted at six, 12, and 24 weeks post surgery, and CRPS-I was diagnosed using clinical criteria outlined by the International Association for the Study of Pain (IASP). The incidence of CRPS-I over a six-month period was significantly lower among patients in group 3 (p = 0.02). Therefore, the authors suggest considering preventive VC therapy whenever possible for individuals undergoing SaSS.

Osteoarthritis

The involvement of free radicals in contributing to the damage associated with osteoarthritis (OA) has long been a focus of research. Several studies, including a cross-sectional examination of knee OA, have suggested that levels of antioxidants in joint fluid are lower in individuals with severe arthritis compared to those with healthy cartilage [15]. This finding further highlights the potential role of free radicals in the development of OA. Given this understanding, the use of ascorbic acid as a preventive measure against the onset or progression of OA has garnered significant research interest.

To date, according to Meacock et al., experimental OA studies in guinea pigs have shown an association between vitamin C supplementation and a reduction in the development of spontaneous OA but no significant difference in preventing lesions after inducing OA [16].

In a 2011 human study by Peregoy and Wilder, the effects of ascorbic acid supplementation on the incidence and progression of knee osteoarthritis were investigated [17]. The study provided a nuanced perspective on the potential role of ascorbic acid in knee OA. While no evidence was found to support a protective role in the progression of existing knee OA, the data, after adjusting for confounding variables, suggest that ascorbic acid supplementation might be beneficial in preventing the occurrence or onset of knee OA. This suggests that ascorbic acid may have a more preventive rather than curative effect in the context of knee OA. It underscores the importance of considering various factors and confounding variables when interpreting the effects of ascorbic acid on knee health. Further research and well-controlled studies would be necessary to clarify the specific mechanisms and conditions under which ascorbic acid supplementation may offer preventive benefits for knee OA.

Osteoporosis

Many studies have shown a correlation between vitamin C and the risk of fractures. A study by Sun et al. assessed the relationship between antioxidant intake and hip fracture risk in the elderly Chinese population [18]. The study involved 726 subjects with hip fractures and the same number of control subjects. Using a food frequency questionnaire and face-to-face interviews, the researchers evaluated various antioxidant intakes and found a significant inverse correlation between dietary ascorbic acid intake and hip fracture risk. However, Sun et al. also analyzed potential limitations such as recall bias and assumptions in estimating food antioxidant values due to possible dietary changes [18].

Similarly, Torbergsen et al. conducted a study concluding that lower levels of ascorbic acid were associated with an elevated chance of hip fracture in older subjects, accounting for its involvement in bone turnover mechanisms [19]. These findings underscore the importance of considering both dietary factors and serum ascorbic acid levels in understanding and mitigating hip fracture risk among the elderly population.

In a recent trial conducted by Sandukji et al., the impact of antioxidants, including ascorbic acid, on oxidative stress and bone healing following long-bone fixative surgery was examined [20]. The study enrolled 55 subjects who were divided into four groups. Groups 1 and 2 received standard postoperative treatment, while groups 3 and 4 were administered a daily antioxidant tablet containing vitamin A, vitamin E, ascorbic acid, and selenium for either one or two weeks. The results indicated that both one and two weeks of postoperative antioxidant supplementation resulted in a notable decrease in oxidative stress markers compared to the control group. However, the authors cautioned about potential limitations, including a small sample size and the inability to discern the effects of individual antioxidants. Despite these considerations, the study suggests a potential beneficial impact of antioxidant supplementation, including ascorbic acid, in alleviating oxidative stress and promoting bone healing following long-bone fixative surgery.

Tendon healing

Tendon injuries present a significant challenge for both active and sedentary populations [21]. These injuries frequently entail the tearing or swelling of tendons, like those found in the biceps, rotator cuff, Achilles, or patella [22]. Collagen, which makes up approximately 75% of the dry weight of tendons, is crucial for their structure [23]. Consequently, interventions involving nutrition and exercise have been suggested to promote collagen synthesis, aiming to strengthen tendons and reduce the risk of injury [24].

In a study by Morikawa et al., the impact of locally injected ascorbic acid on rotator cuff tendon degeneration in mice was investigated [25]. Over an eight-week period, mice with induced rotator cuff degeneration were split into two groups: one given injections of distilled water and the other receiving injections of water supplemented with ascorbic acid. The findings indicated that the group receiving ascorbic acid showed reduced histological changes compared to the control group, suggesting that ascorbic acid may have a preventive effect on oxidative stress-induced rotator cuff degeneration. These results highlight the potential role of ascorbic acid in addressing tendon degeneration and may have implications for interventions aimed at mitigating tendon-related issues.

Anterior cruciate ligament (ACL) reconstruction and knee arthroplasty

The anterior cruciate ligament (ACL) is the knee's most frequently injured ligament, resulting in over 100,000 ACL reconstructive surgeries performed annually in the United States [26]. Research indicates that people who undergo ACL reconstruction surgery might face a higher likelihood of developing osteoarthritis (OA) in the years after the operation, with OA mainly impacting the knee joint [27].

The potential of ascorbic acid to counteract oxidative stress highlights its significance in exploring therapeutic options for individuals undergoing ACL reconstruction and those at risk of OA [28].

In a study conducted by Barker et al., the effects of preoperative ascorbic acid supplementation on inflammation following ACL reconstruction were examined [29]. The study included 20 patients undergoing ACL surgery, who were randomly divided into two groups: the antioxidant group (receiving 500 mg of ascorbic acid and 200 IU of vitamin E twice daily) and the placebo group. Over a span of 12 weeks, the researchers observed that antioxidant supplementation helped maintain plasma ascorbic acid levels and decreased the rise in a pro-inflammatory cytokine 90 minutes after the surgery. Additionally, they noted that antioxidant supplementation led to a more significant improvement in strength in the injured limb at the 12-week mark compared to the control group, though this difference did not reach statistical significance. These findings suggest a potential role for ascorbic acid supplementation in modulating the inflammatory response and aiding in strength recovery following ACL reconstruction.

Behrend et al. examined the effects of perioperative ascorbic acid supplementation on knee range of motion and the risk of arthrofibrosis (AF) after total knee arthroplasty (TKA) [30]. The study involved 95 TKA patients who were divided into two groups: a placebo group receiving a daily placebo pill and an ascorbic acid group receiving 100 mg of oral ascorbic acid daily. Supplementation began the day before the procedure and continued for 50 days. The authors observed that ascorbic acid serum concentration decreased following TKA in the placebo group but not in the ascorbic acid group. They also found that patients experiencing drops greater than 30 μmol/L were more likely to develop AF at one year. The study's limitations comprised a small sample size and a failure to measure inflammation-related biomarkers.

Similarly, Shah et al. examined changes in ascorbic acid and inflammatory cytokine levels in TKA patients [31]. Blood samples from 10 patients were collected pre- and postoperatively. The authors observed a significant increase in inflammation but no significant changes in serum ascorbic acid levels following TKA. They acknowledged limitations such as a small sample size and a short study period.

The precise role of ascorbic acid following total knee arthroplasty (TKA) and ACL reconstruction is not yet fully comprehended, and the available evidence endorsing its perioperative utilization in knee joint procedures remains restricted. Additional research is imperative to clarify the potential advantages of ascorbic acid supplementation in improving outcomes post-ACL reconstruction, lowering the incidence of osteoarthritis (OA), and averting arthrofibrosis (AF). More comprehensive human trials are needed to validate the decrease in ascorbic acid levels and investigate its purported benefits following TKA.

Spinal pathologies

In a study by Lee et al., they prospectively analyzed 123 patients who underwent single-level posterior lumbar interbody fusion (PLIF) and were followed up for at least one year [32]. The patients were divided into two groups: Group A received ascorbic acid (62 patients), and group B received a placebo (61 patients). The Oswestry Disability Index (ODI) scores of group A at three months postoperatively were significantly higher than those of group B (p = 0.04) [33]. Similarly, the ODI scores of group A were slightly higher than those of group B after six postoperative months. These findings suggest that ascorbic acid may be associated with improved functional status after surgery, particularly during the first three postoperative months.

Bone healing

The advancement of specific and sensitive biochemical markers, such as osteocalcin and alkaline phosphatase, has significantly improved the assessment of bone turnover in various metabolic bone diseases [34]. These biomarkers are vital for monitoring the effectiveness of treatments for conditions such as bone fractures and osteoporosis [35]. Studies involving experimental animals have shown that elevated levels of osteocalcin contribute to accelerated bone fracture healing, as evidenced by biochemical, immunohistochemical, histological, and radiological evaluations of callus formation.

In a study, a significant increase in plasma osteocalcin levels was observed in patients treated with antioxidants over a two-week period compared to those in the non-antioxidant-treated group. Following long-bone fixative surgeries, bone markers, including osteocalcin levels and alkaline phosphatase activity, notably increased after two weeks of antioxidant administration. However, osteocalcin levels did not show significant changes after one week of antioxidant supplementation compared to values in the non-antioxidant-supplemented group. Furthermore, lipid peroxidation levels, measured as thiobarbituric acid reactive substances (TBARS), significantly decreased after the administration of antioxidants for one and two weeks compared to those in the non-antioxidant groups. The study illustrated a substantial increase in glutathione (GSH) levels in patients following antioxidant administration, although this effect was notably observed only after two weeks of treatment. These findings collectively suggest that antioxidant supplementation positively impacts bone health and oxidative stress markers, highlighting the potential benefits of antioxidant interventions in bone healing and postsurgical recovery contexts.

Wound healing

Classically, complications arising from a deficiency in ascorbic acid (AA) are most commonly observed in patients with scurvy; however, contemporary individuals often do not exhibit these characteristic symptoms. Those particularly susceptible to AA deficiency include individuals dealing with vascular disease, the elderly, pregnant females, smokers, substance abusers, and malnourished individuals. This vulnerability is supported by the following four reported cases, where all patients faced an increased risk of developing an AA deficiency, subsequently leading to suboptimal wound healing. The administration of AA as a treatment resulted in swift improvements in wound healing for these patients. Ascorbic acid (AA) levels were quantified using high-performance liquid chromatography (HPLC) with ultraviolet (UV) detection (recipe) in a cohort of surgical patients at a teaching hospital (n = 180), either presurgery or post surgery within a week. Over a period of 21 months, suboptimal AA levels, falling below the reference threshold of 25 μmol/L, were identified in 65 out of 180 patients (36%). A detailed description of four patients initially highlighted compromised wound healing, with recorded AA levels of 8, 4, and 19 μmol/L, respectively. Following the initiation of supplementation at a dosage of 1,000 mg/day, a remarkable and rapid recovery of extensive and intricate wounds was noticeable to both patients and their attending clinicians. Supplementation was discontinued upon the complete healing of the wounds [36].

Burns

Precision in fluid resuscitation for burn patients is crucial for optimizing clinical outcomes, with the primary goal being to ensure adequate perfusion to vital organs while minimizing the risks of fluid creep and over-resuscitation. Excessive fluid administration can lead to various complications, including pulmonary and cerebral edema, as well as compartment syndromes. The increased capillary permeability following a burn is largely attributed to the excessive production of inflammatory mediators and the resulting surplus of reactive oxygen species (ROS), which induce endothelial damage to cell membrane lipids and proteins [37,38].

In a retrospective review by Kahn et al. in 2011, ascorbic acid infusion (66 mg/kg/hour) in burn patients with >20% total body surface area (TBSA) was found to reduce overall resuscitation fluids in the first 24 hours by approximately 25% (p < 0.05) [39]. No increased risk of renal failure was reported, but there was no observed improvement in respiratory function or mortality rate.

Another randomized controlled trial by Tanwar et al. in 2018 concluded that administering high-dose ascorbic acid (66 mg/kg/hour) as an adjuvant therapy in resuscitating burn patients in the first 24 hours reduced fluid requirements in the first 24 hours (p < 0.001) for burn patients with >35% TBSA [40]. This high dose of ascorbic acid increased urine output (p = 0.006), decreased fluid retention in the body (p = 0.046), and lowered malondialdehyde levels, indicating the antioxidant effect of ascorbic acid in burn patients.

## Conclusions

The investigation into ascorbic acid's role in molecular pathways associated with bone and tendon regeneration represents a relatively new area of musculoskeletal research. Notably, ascorbic acid supplementation has demonstrated associations with improved functional outcomes, decreased postoperative pain, and a reduced risk of developing complex regional pain syndrome (CRPS) following orthopedic procedures. These findings suggest a potential role for ascorbic acid in enhancing the recovery and outcomes of orthopedic interventions.

However, it is essential to highlight the necessity for high-quality human trials to establish definitive evidence regarding the efficacy of ascorbic acid in enhancing the outcomes of orthopedic procedures. Such trials would help confirm the proposed benefits of ascorbic acid and provide insights into optimal dosages and methods of administration. Rigorous research in this domain is crucial for informing clinical practices and ensuring that ascorbic acid supplementation is effectively utilized to promote musculoskeletal health and recovery in orthopedic settings.
